# Kidney Salvage with Renal Artery Reconstruction after Blunt Traumatic Injury

**DOI:** 10.1155/2020/6162158

**Published:** 2020-03-15

**Authors:** David G. Jackson, Phillipe Abreu, Manuel Anthony Moutinho, Antonio Marttos, George W. Burke, Nicholas Namias, Gaetano Ciancio

**Affiliations:** ^1^Department of Surgery, University of Miami Miller School of Medicine, USA; ^2^Multi-Organ Transplant Program, University of Toronto, Canada; ^3^Ryder Trauma Center, Jackson Memorial Hospital, University of Miami Miller School of Medicine, USA; ^4^Miami Transplant Institute, Jackson Memorial Hospital, University of Miami Miller School of Medicine, USA

## Abstract

Renal artery injury from blunt abdominal trauma is a rare condition that is typically managed nonoperatively in hemodynamically stable patients. Revascularization can be achieved by stenting or surgical reconstruction of the renal artery. All attempts at revascularization should minimize warm ischemic time. Here, we discuss a patient postmotor vehicle accident who presented to Ryder Trauma Center with intra-abdominal bleeding. He underwent emergency exploratory laparotomy with splenectomy and abdominal packing. Postoperative CT scan revealed a contrast nonenhancing left kidney. The patient then returned to the operating room and underwent in situ renal artery reconstruction after >4 hours of warm ischemia. The patient survived a 2-month hospital course and was discharged home after prolonged in-hospital stay and intensive care treatment. Nuclear medicine scan showed scarring and atrophy of the reattached kidney with 16.3% of overall function attributed to the affected kidney. This case shows that patients with renal artery injury can be managed operatively with arterial reconstruction. Reducing warm ischemic time is critical in preserving kidney function.

## 1. Introduction

One rare consequence of blunt abdominal trauma is renal artery injury (incidence 0.05-0.1%) [1, 2]. Awareness of blunt renal artery trauma has increased due to improved detection by CT scan [3]. Blunt renal artery trauma can cause renal artery aneurysm, dissection, or rupture with subsequent renal infarction [4]. Renal trauma is graded on a spectrum (Grades I-V) [5]. Management of blunt renal artery injury can be nonoperative or operative, with the latter usually accomplished through endovascular stenting [6]. Nonoperative vs. operative management of blunt renal trauma depends on the severity of the trauma and laterality of the injury [1].

Conservative approaches are typically attempted in patients with unilateral renal injury who do not undergo exploratory laparotomy, while bilateral renal injury necessitates surgical revascularization. In cases with complications such as renal artery thrombosis, surgical revascularization is essential [7]. If the patient is unstable, more lethal injuries should be ameliorated prior to renal revascularization [1]. Even after revascularization, the patient may experience posttraumatic acute kidney injury (AKI) from renal crush injury and hemorrhagic shock [8].

Many revascularization attempts have been unsuccessful, with 26% of success rate as published by Haas and Spirnak [9]. The incidence of chronic complications such as decreased renal function and hypertension depends on factors including age, comorbidities, length of ischemic time, and prehospital trauma life support. Here, we present a patient who sustained blunt renal artery injury and underwent surgical revascularization at our institution.

## 2. Case Report

A 48-year-old male with no significant past medical history who was a restrained driver in a motor vehicle accident sustained multiple injuries in the crash (Table 1) and had a prolonged and difficult extrication from the crash site before being brought to Ryder Trauma Center with a Glasgow Coma Scale of 15 approximately 1-2 hours after his accident. While in the resuscitation bay, bilateral chest tubes were placed for bilateral pneumothoraces and he had a positive FAST exam with tachycardia and severe hypotension (HR 128, BP 69/50). He was immediately brought to the operating room (OR) for emergency exploratory laparotomy, and splenectomy was performed for Grade V splenic injury, followed by abdominal packing without complete abdominal closure. His condition stabilized intraoperatively; no active abdominal bleeding or hematoma could be identified at that moment.

**Figure 1:**
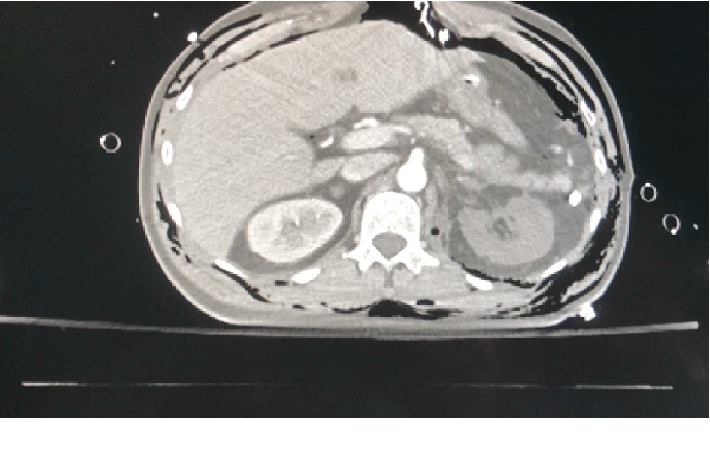
CT scan with contrast showing nonenhancing left kidney consistent with left renal artery injury.

A full-body CT scan was then requested to investigate other injuries. A list of numerous bone fractures that demonstrate the serious extent of the traumatic injury is shown in Table 1. The patient had also a scalp injury and needed additional workup to rule out traumatic brain injury. Head CT was negative. Chest CT demonstrated no further injuries other than minor pulmonary contusions and pneumothoraxes already being treated by the previously placed chest tubes. Abdominal contrasted CT identified a left renal artery injury and a nonenhancing ischemic left kidney but no other abdominal organ injuries (Figure 1). In an attempt to salvage left kidney function despite over 4 hours of warm ischemia at that point, the patient was taken back to the OR for emergent repair of his left renal artery/in situ autotransplantation.

**Figure 2:**
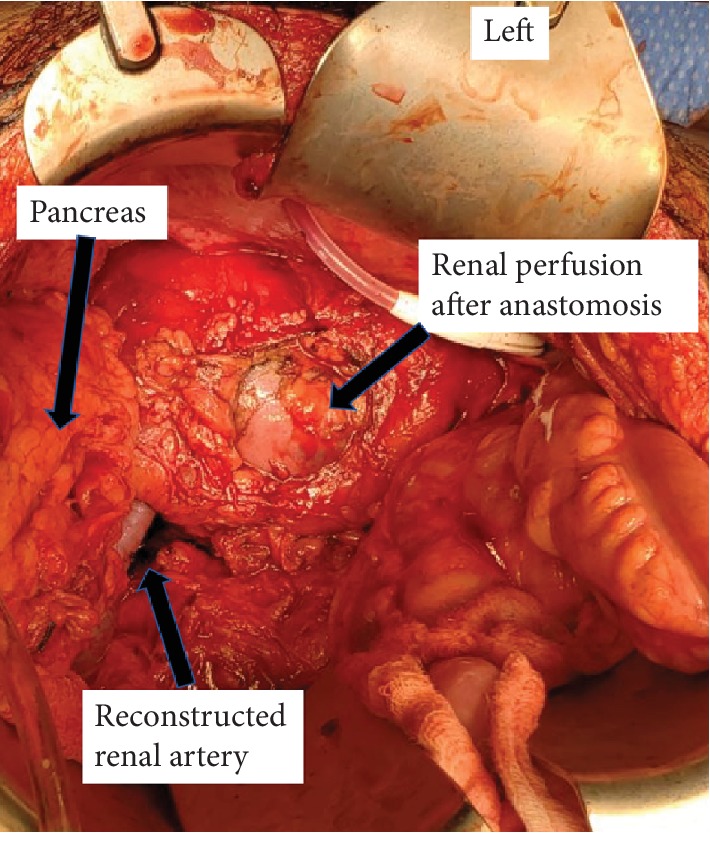
Surgical photography demonstrating renal parenchymal perfusion after reconstruction and anastomosis.

An occlusive 2 cm intra-arterial thrombus was removed, and a corresponding 2 cm damaged segment of the renal artery was excised. The kidney was flushed through the renal vein with histidine-tryptophan-ketoglutarate perfusion solution during the anastomosis of the renal artery. The left kidney was adequately perfused postreconstruction of the left renal artery (Figure 2).

During the two operations, the patient received a total of 7 units of packed red blood cells, 6 units of fresh frozen plasma, 2 units of cryoprecipitate, and 2 platelet transfusions. The patient was subsequently admitted to the Trauma Intensive Care Unit (TICU) for postoperative care. His hospital course was significant for worsening azotemia in the setting of decreased kidney function and falling urine output with a peak creatinine above 8 mg/dL. Dialysis and pressors were started on postoperative day 4. Eventually, his renal function improved, and his creatinine fell, and urinary output increased. He exhibited hypertension 2-3 weeks postrenal artery repair, but his systolic blood pressures eventually stabilized between 110 and 130 mmHg. He recovered in our TICU after multiple episodes of fungemia and bacteremia, was transferred to the trauma floor, and was discharged two months after admission. He is currently being followed in an outpatient clinic. After 18 months of follow-up, his serum creatinine level is 1.3 mg/dL and vital signs were within normal limits, without hypertension.

**Figure 3:**
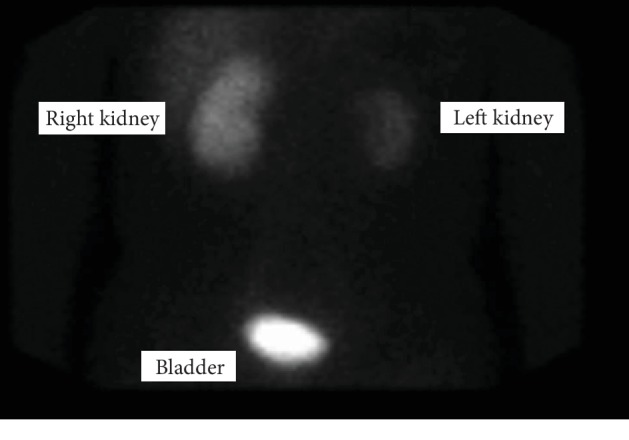
Follow-up nuclear scan (11 mCi technetium-99 m MAG3) demonstrating right kidney perfusion dominance at 6 months postsurgery.

Outpatient nuclear medicine renal scan indicated partial preservation of left renal function with a shrunken left kidney, and 16.3% (Figure 3) of split renal function attributed to the left kidney. Renal biopsy of the revascularized kidney was not performed. Left renal artery stenosis was not noted after its reattachment.

## 3. Discussion

Blunt renal artery trauma can be managed conservatively or surgically [1]. This patient presented with intra-abdominal bleeding and needed an emergent exploratory laparotomy prior to further abdominal investigation. Our patient's renal artery injury was severe. This is in contrast to what renal artery injury patients have who generally had minor renal artery injuries and underwent conservative management [1, 2, 10]. In a cohort published by Sangthong et al., only 8.7% of patients underwent operative renal artery reconstruction [2]. The open operative technique we used (in situ renal artery reconstruction) was also unlike most operative management of renal artery trauma treated elsewhere with endovascular approach [6]. In this case, endovascular repair would very likely not have been possible due to the degree of damage to the renal artery. However, where endovascular treatment is not available for trauma patients, open repair must be considered an option for the treatment of arterial injuries.

Thrombosis of end arterial supply is an uncommon indication for exploratory laparotomy in trauma, followed by renal artery surgical repair [7]. Our patient had two episodes of postoperative AKI. This may have resulted from prolonged ischemia to both kidneys due to hemorrhagic shock after difficult extrication and delayed prehospital trauma life support [8]. Salvage of an affected kidney may be of lower priority than more critical injuries including severe splenic hemorrhage and other abdominal organ injuries leading to major bleeding [1]. Renal artery injury bleeding may be contained and controlled within Gerota's fascia. We attempted renal revascularization only after his hemodynamic condition was stabilized. Surgical revascularization should be attempted soon after hemodynamic stabilization to minimize warm ischemic time. In addition, the patient would have to return to the OR after CT was performed at the least to remove abdominal packing.

This patient was initially managed in the TICU, which is the common management for up to 70% of blunt renal artery injury patients [2]. The intraoperative revascularization of the renal artery was however associated with longer ICU stay. Renal artery injury patients may develop hypertension as a long-term consequence of their injury/repair [9]. This patient sustained other injuries that caused him significant associated morbidity and pain. His blood pressure will need to be followed closely in order to diagnose and treat possible hypertension that may follow this injury to the left kidney.

An important question would be whether 16.3% of renal function at follow-up could be justified as acceptable renal salvage and justify the approach used in our case. The salvage of this kidney via arterial reconstruction was performed in order to optimize the patient's total physiologic capacity; while the degree of total functional return could not be estimated at the time, we felt that this justified the approach used in our case. Indeed, the question whether there is an upper limit of ischemic/traumatic injury beyond which a kidney should be considered unsalvageable remains incompletely answered [11]. The potential harm of a renal artery reconstruction should be weighed against the loss of function after arterial thrombosis or even nephrectomy. Better results in terms of long-term kidney function may be achieved in patients with lower prehospital trauma life support time and faster transportation to definitive care. Although clinically less harmful for young patients, radical nephrectomy was associated with double the loss of eGFR and 2.2-fold increased mortality when compared with partial nephrectomy in CKD patients, so radical nephrectomy may carry its own factors that increase mortality [12]. On the other hand, data from renal transplant donors demonstrates that risk of end-stage renal disease after surgically removing the kidneys was comparable to the general population [13].

The present case demonstrates that open operative renal artery reconstruction with autotransplantation is a viable method for managing severe blunt renal artery trauma with associated thrombosis and that special attention must be paid to warm ischemic time leading to posttraumatic AKI [1, 8].

## 4. Conclusion

Patients with high-grade renal injury and thrombosis of the affected renal artery can be managed with resection of the damaged portions of the renal artery and in situ arterial reconstruction of the affected kidney. Caution must be taken to minimize excessive warm ischemic time that would exacerbate postoperative AKI. Patients may have long-term loss of function of the revascularized kidney and/or hypertension.

## Figures and Tables

**Table 1:** List of Injuries sustained by the patient during MVA. CT scan aided in the diagnosis of many of these injuries.

(1) Pulmonary contusions and pneumomediastinum
(2) Bilateral pneumothoraces
(3) Multiple bilateral rib fractures
(4) Manubrium fracture
(5) Bilateral clavicle fractures
(6) Left scapula fracture
(7) Multiple cervical spine fractures: left occipital condyle fracture, left C1 facet fracture, left C2 lateral mass fracture, and left C6-C7 laminar fracture
(8) Cortical irregularity at the superior endplates of T1, T3, T4, and T5 likely representing compression fractures
(9) Left T1 transverse process fracture
(10) Minimally displaced T3 and T7 spinous process fractures
(11) Comminuted displaced basicervical fracture of the right proximal femur, underwent open reduction internal fixation with proximal tibia traction pin
(12) Comminuted displaced subtrochanteric fracture of the left proximal femur, underwent open reduction internal fixation
(13) Extensive subcutaneous emphysema
(14) Scalp injury and suspected traumatic brain injury upon admission
(15) Noncontrast enhancing left kidney
